# Impacts of post-transplantation cyclophosphamide treatment after allogeneic hematopoietic stem cell transplantation in acute myeloid leukemia

**DOI:** 10.1038/s41598-019-38644-1

**Published:** 2019-02-14

**Authors:** Sinem Namdaroglu, Ali Hakan Kaya, Hikmettullah Batgi, Omur Kayikci, Mehmet Sinan Dal, Dicle Iskender, Merih Kizil Cakar, Emre Tekgunduz, Fevzi Altuntas

**Affiliations:** Department of Hematology, Stem Cell Transplantation Unit, University of Health Sciences,Ankara Oncology Training and Research Hospital, Ankara, Turkey

## Abstract

Post-transplant cyclophosphamide has become a promising medical option after allogeneic HSCT. In this study we aimed to evaluate the efficacy of cyclophosphamide and cyclosporine combination in acute and chronic graft-versus-host disease (GvHD) prophylaxis in acute myeloid leukemia (AML) cases scheduled for allogeneic hematopoietic stem cell transplantation (allo-HSCT). Retrospective analysis of data from 40 cases who underwent allogeneic HSCT under GvHD prophylaxis with cyclophosphamide and cyclosporine combination between April 2016 and August 2017 was made. Cyclophosphamide was given at daily doses of 50 mg/kg on post-transplant 3^rd^ and 4^th^ days, and cyclosporine was applied at daily doses of 3 mg/kg/day starting from the 5^th^ post-transplant day. Cyclosporine dose was tapered beginning from the 45^th^ postoperative day and completely discontinued on the 90^th^ post-transplant day. Mean age was 38.25 ± 15.25 years. Posttransplant median follow-up was six months (6–17 months). Post-transplant, the number of deaths and mortality rates related and unrelated to transplantation were 5 (12.5%), and 2 (5%), respectively. Acute GvHD was diagnosed in 7 cases (17.5%), and relapse was noted in 9 cases (22.5%). Myeloablative or reduced intensity conditioning was performed in 22 (55%) and 18 (45%) patients, respectively. The distribution of the donors was as follows: match-related (n = 26; 65%), match-unrelated (n = 9, 22.5%) and haploidentical donors (n = 5; 12.5%). There was no statistically significant correlation between the transplant-related and unrelated mortality and parameters under investigation.Cyclophosphamide use appears to be a highly effective and promising strategy for acute GvHD prophylaxis in non-haploidentical allogeneic HSCT cases. Identification of the impact of cyclophosphamide use on the development of chronic GvHD needs further investigation.

## Introduction

Transplantation of hematopoietic stem cells from any source (bone marrow, peripheral blood, umbilical cord blood) is a treatment not only for hematopoietic system diseases but also for metabolic and immunological disorders. Patients with hematopoietic stem cell transplantation (HSCT) carry a high risk of transplant-related mortality and morbidity due to immunological mechanisms, toxicity due to drugs used in the preparation regimens, and long hospitalization times^1^. In addition to early complications of HSCT, particularly allogeneic transplant patients are exposed to long-term consequences that require lifelong follow-up and treatment^2^.

Transplantation-related mortality has gradually decreased in recent years with the development of supportive therapies, preventive treatments, and early diagnosis facilities. However, HSCT, in addition to its therapeutic properties, faces us with its many complications. HSCT can be classified according to the source of the progenitor cell used. Although both of these sources have advantages and disadvantages, both infectious and non-infectious complications are more likely to occur in allogeneic transplantations^1,2^. In a cohort study involving 1479 patients with at least two years survival after allogeneic HSCT, relapse of the primary disease was the most frequent cause of mortality (29%), while the most common causes of non-relapse mortality were graft-versus-host disease (GvHD) (22%), infections (11%), secondary malignancies (7%), pulmonary complications (5%), cardiac toxicity (2%) and other treatment-related events (8%)^3^.

In a similar retrospective analysis performed in autologous stem cell transplantation treated diffuse large β cell patients, causes of mortality apart from relapse of the disease were found to be respiratory failure (31%), infections (13%), cardiac toxicity (15%) and secondary malignancies (15%)^4^. Allogeneic HSCT is a treatment option with the potential to cure many malignant and non-malignant hematological disorders. As results are improved with preventive and supportive therapies, indications are also developing. Alternative stem cell sources have increased the likelihood of finding donors, and even haploidentical transplantation have yielded acceptable, successful outcomes.

Post-transplant cyclophosphamide has become a promising medical option after allogeneic HSCT. This method has gained popularity due to its safety profile and efficacy for reduction of GvHD^5^. HLA-haploidentical HSCT using post-transplant high-dose cyclophosphamide is becoming a more popular alternative strategy for allogeneic HSCT^6^.

The aim of the present study was to investigate the effect of posttransplant cyclophosphamide use on mortality, relapse and acute or chronic GvHD formation in acute myeloid leukemia (AML) patients with allogeneic HSCT.

## Materials and Methods

### Study design

In this retrospective study, data were extracted from the files of 40 AML patients treated with allogeneic HSCT in a tertiary center who were also receiving immunosuppressive therapy with cyclophosphamide and cyclosporine during the post-transplant period. This study was conducted by the Declaration of Helsinki and was approved by Ankara Oncology Training and Research hospital ethics committee. Written informed consent was obtained from all patients.

In our center, medical records of allogeneic HSCT patients under prophylaxis for GvHD with cyclophosphamide during the post-transplant period were retrospectively evaluated between April 2016 and August 2017. A total of 40 patients (14 female, 26 male) had a mean age of 38.25 ± 15.25 years in our series. In all cases, cyclophosphamide at daily doses of 50 mg/kg was given on 3^rd^ and 4^th^ days after transplantation, and cyclosporine at daily doses of 3 mg/kg/day starting from the 5^th^ postoperative day was administered. Cyclosporine dose was tapered beginning from the 45^th^ postoperative day, and completely discontinued on the 90^th^ postoperative day. Acute GvHD detected on the cases was staged according to standard criteria.

Patients were divided into two groups according to pre-transplant risk class, chemotherapy regimen applied (myeloablative or reduced intensity monitoring), date of transplantation, GvHD, the presence of a genetic anomaly, transplant-related and unrelated 100-day mortality rates and relapses were recorded.

Cytogenetic risk classification was performed according to standard criteria^7^. Conditioning intensity was defined as myeloablative or reduced intensity using the consensus criteria^8^. The day of platelet recovery was termed as the first day on which the platelet count reached or exceeded 20 × 10^9^/L without transfusion. The day of neutrophil recovery was defined as the first of 3 consecutive days on which the absolute neutrophil count was more than 0.5 × 10^9^/L. The International Bone Marrow Transplant Registry criteria were used for acute GvHD staging and grading, and the National Institute of Health criteria were used to determine chronic GvHD severity^9–11^.

Initial neutrophil engraftment was defined as the first of 3 consecutive days with an ANC of more than 0.5 × 10^9^/L. Platelet engraftment was defined as the first of 3 consecutive days with a PLT count of more than 20 × 10^9^/L without transfusion support. GVHD was graded by the consensus criteria^12–14^.

The inclusion criteria for this study consisted of: (1) using G-CSF mobilized peripheral blood as graft source, (2) using a matched related/unrelated or haploidentical donor and (3) using PT-Cy (50 mg/kg on days +3 and +4), a calcineurin inhibitor and mycophenolate mofetil as GvHD prophylaxis. Filgrastim 5 μg/kg daily was administered starting day +5 until neutrophil recovery. The lack of complete remission on the last bone marrow biopsy performed within 4 weeks before conditioning initiation was accepted as active disease.

Donor engraftment was determined by a PCR-based assay for STRs from bone marrow samples or peripheral blood mononuclear cells^9^, and defined as donor chimerism >95%, and graft failure as <5% not due to relapse.

Graft failure, relapse or death (all without GvHD) were considered competing risks for GvHD; relapse was considered a competing risk for nonrelapse mortality; death without relapse was considered competing risk for relapse; and death without count recovery was considered a competing risk for count recovery.

The allogeneic stem cell transplantation was performed with a conditioning regimen including Busulfan s3.2 mg/kg/day for three days (BU_3_), Fludarabine 40 mg/m²/day for four days (FLU_4_)_,_ rabbit Anti-thymocyte globulin 2.5 mg/kg/day for three days.(ATG). Patients were hydrated starting fron 24 hours prior to the stem cell infusion. Premedication consistent of chlorfenoxamine hydrochloride (10 mg) in 100 ml of isotonic saline and dexamethasone (8 mg) in 100 ml isotonic saline was administered within 30 minutes approximately 1 hour before stem cell infusion. The infusion volume and the number of stem cells (CD34+ or nucleated cells) were noted. Irradiation or the uses of infusion pump or leukocyte filter were omitted. The rate of stem cell infusion was 2 ml/min. In case of the absence of any reaction in 30 minutes, infusion was carried on at a rate of 150–200 ml/hour. The infusion is completed maximally within 4 hours. In case of major incompatibility, stem cell transfusion was initiated at a rate of 20 ml/hour in the first 30 minutes and at a rate of 40 ml/hour in the second 30 minutes. After the first hour, infusion was carried on at a rate of 150–200 ml/hour. For major incompatibility, the rates of infusion were 60 ml/hour and 90 ml/hour for the first and second 30 minutes, respectively. After the first hour, the rate was kept at 150–200 ml/hour.

### Statistical analysis

SPSS 21 program (*Chicago, USA*) was used for data analysis. As a descriptive analysis, the mean ± standard deviation was given when assumptions were met for numerical variables, and when not met the median and the smallest-largest values were given. For categorical variables, numerical and percentage values were given. The t-test was used in independent groups when assumptions were met in intergroup comparisons of numerical measurements, when not met the Mann-Whitney U test was used. For the comparisons between the categorical variables, the chi-square test was used. A value of p < 0.05 was considered statistically significant.

## Results

### Posttransplant median follow-up was 6 (range, 1 to 17) months

Most frequently detected genetic anomalies encountered in this series combined WT1 (n = 9, 22.5%), FLT3 ITD and WT1 positivity (n = 4; 10%). Myeloablative or reduced intensity conditioning was performed in 22 (55%) and 18 (45%) patients, respectively. The distribution of the donors was as follows: (MRD) (n = 26; 65%), match-unrelated (n = 9, 22.5%) and haploidentical (n = 5; 12.5%).

As for the number of posttransplant deaths, the mortality rates related, and unrelated to bone marrow transplantation were determined as 5 (12.5%) and 2 cases (5%), respectively.

Acute GvHD has been diagnosed in 7 cases (17.5%) as skin, liver, and gastrointestinal tract involvement can occur. Chronic GvHD was not observed in any case. Relapse was seen in 9 cases (22.5%).

Correlation analysis between parameters studied (aGvHD, relapse, gender, genetic anomaly, pre-transplant protocol, type of donor) and mortality status related or unrelated to transplantation on the 100^th^ postoperative day revealed no statistically significant difference (Tables 1 and 2).

**Table 1:** Correlation between parameters studied and mortality status related to transplantation on the 100^th^ postoperative day.

Variable		Mortality status on the 100^th^ postoperative day related to transplantation		p-value
		Survive (n, %)	Dead (n, %)	
aGvHD	Present	28 (71.8)	4 (10.2)	1.000
	Absent	6 (15.4)	1 (2.6)	
Relapse	Present	26 (66.7)	5 (12.8)	0.570
	Absent	9 (23.1)	0	
Gender	Female	12 (30.8)	2 (5.1)	1.000
	Male	23 (59)	3 (7.7)	
Genetic anomaly	Present	4 (10.2)	0	1.000
	Absent	30 (76.9)	5 (12.8)	
Pre-transplant protocol	Myeloablative	18 (46.2)	4 (10.2)	0.355
	RIC	17 (43.6)	1 (2.6)	
Type of donor	MRD	24 (61.5)	2 (5.1)	0.220
	Haploidentical	3 (7.7)	2 (5.1)	
	MUD	8 (20.5)	1 (2.6)	

(Abbreviations: aGvHD: acute graft versus host disease; RIC: reduced intensity conditioning; MRD: match related donor; MUD: match-unrelated donor).

**Table 2:** Correlation between parameters studied and mortality status unrelated to transplantation on the 100^th^ postoperative day.

Variable		Mortality status on the 100^th^ postoperative day unrelated to transplantation		p-value
		Survive (n, %)	Dead (n, %)	
aGvHD	Present	28 (71.8)	4 (10.2)	1.000
	Absent	6 (15.4)	1 (2.6)	
Relapse	Present	26 (66.7)	5 (12.8)	0.404
	Absent	9 (23.1)	0	
Gender	Female	12 (30.8)	2 (5.1)	0.533
	Male	23 (59)	3 (7.7)	
Genetic anomaly	Present	4 (10.2)	0	1.000
	Absent	30 (76.9)	5 (12.8)	
Pre-transplant protocol	Myeloablative	18 (46.2)	4 (10.2)	0.196
	RIC	17 (43.6)	1 (2.6)	
Type of donor	MRD	24 (61.5)	2 (5.1)	0.301
	Haploidentical	3 (7.7)	2 (5.1)	
	MUD	8 (20.5)	1 (2.6)	

(Abbreviations: aGvHD: acute graft versus host disease; RIC: reduced intensity conditioning; MRD: match related donor; MUD: match-unrelated donor).

Correlation analysis between parameters studied (relapse, gender, genetic anomaly, pre-transplant protocol, type of donor) and aGvHD revealed no statistically significant difference (Table 3).

**Table 3:** The correlation between acute graft-versus-host disease (aGvHD), and the parameters analyzed.

Variable		aGvHD		p-value
		+	−	
Relapse	Present	24 (61.5)	6 (15.4)	1.000
	Absent	8 (20.5)	1 (2.6)	
Gender	Female	10 (25.6)	4 (10.2)	0.225
	Male	22 (56.4)	3 (7.7)	
Genetic anomaly	Present	3 (7.7)	1 (2.6)	1.000
	Absent	28 (71.8)	6 (15.4)	
Pre-transplant protocol	Myeloablative	20 (51.3)	2 (5.1)	0.205
	RIC	12 (30.8)	5 (12.8)	
Type of donor	MRD	21 (55.3)	4 (10.2)	0.424
	Haploidentical	3 (7.7)	2 (5.1)	
	MUD	8 (20.5)	1 (2.6)	

(Abbreviations: aGvHD: acute graft versus host disease; RIC: reduced intensity conditioning; MRD: match related donor; MUD: match unrelated donor).

Correlation analysis between parameters studied (gender, genetic anomaly, pre-transplant protocol, type of donor) and relapse revealed no statistically significant difference (Table 4).

**Table 4:** Correlation between relapse and the parameters analyzed.

Variable		Relapse		p-value
		+	−	
Gender	Present	12 (30.8)	2 (5.1)	0.453
	Absent	19 (48.7)	7 (17.9)	
Genetic anomaly	Female	3 (7.7)	1 (2.6)	1.000
	Male	28 (71.8)	7 (17.9)	
Pretransplant protocol	Myeloablative	18 (46.2)	4 (10.2)	0.705
	RIC	13 (33.3)	5 (12.8)	
Type of donor	MRD	19 (48.7)	7 (17.9)	0.243
	Haploidentical	5 (12.8)	0	
	MUD	7 (17.9)	2 (5.1)	

(Abbreviations: RIC: reduced intensity conditioning; MRD: match related donor; MUD: match unrelated donor).

There was no statistically significant relationship between age-related variables and the transplant-related mortality (p = 0.905), the presence of aGvHD (p = 0.304) and relapse (p = 0.483).

## Discussion

Allogeneic HSCT is a complex procedure which is used to treat many hematological and immunological entities. In spite of its therapeutic potential, toxicities linked with its application usually restrict the utility of allogeneic HSCT against high-risk diseases or if other less toxic therapies have failed. There are mainly two complications related to the procedure: conditioning regimen toxicity and immunological complications^15^. The development of immunological co-morbidities after allogeneic HSCT is related to the diversity in human leukocyte antigen (HLA) status between the donor and the recipient. Efforts have been spent to improve HLA donor typing and use of immunosuppressive agents following allogeneic HSCT.

There is an increasing interest in the use of agents such as cyclophosphamide and cyclosporine for GvHD prophylaxis, both in the haploidentical and HLA-identical settings. This method offers advantages such as the improved effect on GvHD control without any complex procedures like graft manipulation^5,6^. The safety profile of this method is satisfactory compared to previous protocols, and this is evident not solely from the very low rate of graft failure but also in an acceptable incidence of infective complications. There are still many questions awaiting to be replied related to the ways for improvement of these settings. Even if the use of immunosuppressive agents is feasible, the relatively high rate of aGvHD and vagueness on the incidence of cGvHD constitute important challenges. Therefore, keeping aGvHD rate lower with different immunosuppressive protocols is important. In this aim, the use of cyclosporine together with cyclophosphamide have yielded promising results^16^. Consequently, an acceptable toxicity profile and a fast immune reconstitution must be targeted to establish an immunological platform capable for reduction of relapse after transplantation.

Ruggeri *et al*. evaluated the effect of stem cell source in haploidentical transplantation with posttransplantion cyclophosphamide in 451 patients transplanted for AML and revealed that the use of peripheral blood stem cells increases the risk of acute GVHD, whereas survival outcomes are comparable^17^. Experience using post-transplant cyclophosphamide as GVHD prophylaxis in allogeneic HSCT from matched sibling or unrelated donors is limited and with controversial results. Ruggeri *et al*. analyzed 423 patients with acute leukemia who received cyclophosphamide alone or in combination with other immunosuppressive drugs as GVHD prophylaxis and stated that the addition of other immunosuppressive drugs enhances its effect and reduces the risk of severe cGVHD, reducing mortality and improving survival^18^.

Graft-versus-host disease is a leading cause of non-relapse mortality after allogeneic HSCT^19^. More recently, high dose administration of cyclophosphamide after transplantation has been introduced as an effective method of prophylaxis for GvHD^20^. This protocol is based on the logic that alloreactive donor T lymphocytes can be eliminated, which are induced to proliferate soon after transplant, which induces tolerance. Trials on non-myeloablative conditioning haploidentical HSCT with cyclophosphamide after transplantation displayed obvious tolerability with low rates of GvHD, infection, and mortality unrelated to relapse^16^. The relapse of malignancy is still the predominant cause of treatment failure. To reduce the risk of relapse in patients with high-risk hematologic malignancies, the feasibility of cyclophosphamide after transplantation administration utilizing reduced intensity or myeloablative conditioning regimens can be used. Our results indicated that none of the parameters under investigation were significantly linked with mortality, relapse, and GvHD. Owing to the success of cyclophosphamide after transplantation tolerance in the setting of haplo-HCT, this regime has been extended to the patients who underwent allogeneic HSCT from HLA-matched donors. Acute GvHD is a challenge that must be managed, and its incidence can be reduced using the utility of one or more immunosuppressive agents^21^. In this perspective, we recommend the use of cyclophosphamide and cyclosporine as prophylactic agents for GvHD. Currently, sufficient evidence for its efficacy and safety after transplantation has accumulated under both reduced intensity and myeloablative settings. The timing of transplantation is supposed to be critical for a successful outcome for patients with hematological malignancies. Cyclophosphamide after transplantation allows expansion of the donor pool, making marrow transplantation applicable for patients with stem cell donor. This method is associated with a low risk of complications, even with haploidentical related donors. Factors such as the experience of the center, facilities and the availability of clinical trials must be remembered to set the suitable treatment algorithm. Allogeneic HSCT with cyclophosphamide after transplantation, as an immunological platform, provides the chance to safely explore innovative approaches to diminish the risk of relapse following transplant^5,6^.

An ideal immunosuppressive regimen needs to be effective for patients with standard or high-risk myeloid malignancies, who are going to receive HSCT. It is expected to have reduced extramedullary toxicity, low rate of relapse, acute and chronic GvHD rate, and improved survival.

Stem cell transplantation is an important treatment modality and cure for malignant or non-malignant bone marrow diseases. However, considering the causes of mortality other than the relapse of the primary disease, long-term multidisciplinary and close follow-up of the patients is needed, because of the early or long-term comorbidities and complications that may arise. In a recent study, Chiusolo *et al*. suggested that post-transplant cyclophosphamide regimen provided protection against GVHD, low toxicity, and encouraging low relapse incidence in AML patients who received unmanipulated haploidentical marrow transplants along with a myeloablative regimen^21^. Bacigalupo *et al*. implied that a myeloablative conditioning regimen followed by unmanipulated haploidentical bone marrow transplantation with post-transplant cyclophosphamide was associated with a low risk of acute and chronic GVHD and encouraging rates of mortality and overall survival^22^. Another recent publication demonstrated that an MA conditioning regimen followed by haploidentical bone marrow transplantation with BMT with post-transplant cyclophosphamide resulted in a low risk of acute and chronic GVHD and more promising rates of transplant-related mortality and disease-free survival^23^. Klein *et al*. reported excellent rates of engraftment, GVHD, and TRM in pediatric/young adult patients treated with post-transplant cyclophosphamide regimen. They suggested that this approach was a widely available, safe, and feasible option for pediatric and young adult patients with high-risk hematologic malignancies, including those with a prior history of myeloablative bone marrow transplantation and/or those with comorbidities or organ dysfunction^24^. Thus, the results of relevant contemporary publications are mainly consistent with our findings.

We could not determine any significant relationship between relapse, gender, type of donor, genetic anomaly, pre-transplant protocol and mortality, relapse and GvHD. This may be associated with limitations of our study such as retrospective design, single-centered construct, relatively low number of cases and short-term follow-up. Conduction of multi-centered, controlled, prospective relevant studies with larger series and longer duration of follow-up is needed.

In the last decade, the most important development in AML has been the demonstration of a relationship of some cytogenetic anomalies with prognosis. The cytogenetic properties of the patients are the most important factors for determining the time of transplantation for most leukemia specialists. Follow-up after consolidation is recommended for patients with a good cytogenetic prognostic profile, and those who developed remission after induction therapy. In patients having favorable prognostic criteria, patients with PR or relapses, following induction and consolidation of chemotherapies in patients with moderate or poor prognosis allogeneic HSCT should be performed. If appropriate HLA -matched unrelated donors are available, HSCT should be performed using these donors.

To conclude, cyclophosphamide use appears to be a highly effective and promising strategy for acute GvHD prophylaxis in allogeneic HSCT cases. Due to the short duration of median follow-up, it is too early to make a healthy interpretation concerning the impact of this approach on the development of chronic GvHD, and factors related to mortality and relapse.
